# Raman Spectroscopy of Optically Trapped Living Human T Cell Subsets and Monocytes

**DOI:** 10.3390/ijms25179557

**Published:** 2024-09-03

**Authors:** Martin Nötzel, Maria Mahamid, Romy Kronstein-Wiedemann, Tjalf Ziemssen, Katja Akgün

**Affiliations:** 1 Department of Neurology, Technische Universität Dresden, 01307 Dresden, Germany; 2 Department of Transfusion Medicine, Medical Faculty Carl Gustav Carus, Technische Universität Dresden, Fetscherstraße 74, 01307 Dresden, Germany

**Keywords:** Raman spectroscopy, T cells, CD4, CD8, monocytes, optical trapping, CD3/CD28 stimulation

## Abstract

In recent years, Raman spectroscopy has garnered growing interest in the field of biomedical research. It offers a non-invasive and label-free approach to defining the molecular fingerprint of immune cells. We utilized Raman spectroscopy on optically trapped immune cells to investigate their molecular compositions. While numerous immune cell types have been studied in the past, the characterization of living human CD3/CD28-stimulated T cell subsets remains incomplete. In this study, we demonstrate the capability of Raman spectroscopy to readily distinguish between naïve and stimulated CD4 and CD8 cells. Additionally, we compared these cells with monocytes and discovered remarkable similarities between stimulated T cells and monocytes. This paper contributes to expanding our knowledge of Raman spectroscopy of immune cells and serves as a launching point for future clinical applications.

## 1. Introduction

Immune cell phenotyping and characterization of functional properties play an important role in the diagnostic and therapeutic management of many autoimmune diseases. In order to develop individualized strategies in medical care, novel approaches to improve cellular biomarker monitoring are needed. Raman spectroscopy has emerged as a powerful tool to measure the ‘chemical fingerprint’ of biological matter, such as cells, in a non-invasive and label-free way. Furthermore, there is no need for chemical fixation that might alter the cell’s characteristics. In recent years, Raman spectroscopy has become an interesting candidate for future medical and biomedical diagnostics [1,2].

Raman spectroscopy has been employed in the diagnosis of various types of cancer, such as breast, prostate, and pancreatic cancer. It can distinguish between healthy and cancerous tissue based on differences in the Raman spectra [3]. Furthermore, Raman spectroscopy was utilized for in vivo measurements of water concentration in brain tissue. This research is significant for understanding brain health and conditions like edema, as it allows the non-invasive analysis of water content in brain tissues, a key parameter in neuroscience [4].

While Raman spectroscopy has found extensive application in cancer research for its ability to provide molecular information about tissues, its utilization in the investigation of autoimmune diseases has been comparatively limited [5]. The investigation of CD4 and CD8 cells, along with the examination of activated CD4 and CD8 cells, holds paramount significance in unraveling the fundamental mechanisms of autoimmune diseases such as neuroimmunological or other inflammatory disorders and aiding in their diagnosis. The identification and characterization of these cell types serve as potential diagnostic markers, offering a deeper understanding of the immunological signatures associated with the disease and facilitating more precise diagnostic strategies for individuals affected by severe autoimmune diseases.

Raman spectroscopy has already been applied to some extent in immunology as a powerful tool for investigating the diverse population of immune cells, unraveling intricate connections within the field. T cells, B-cells, monocytes, and macrophages have already been studied [6,7,8,9]. For example, Chen et al. have succeeded in distinguishing CD4 and CD8 cells using Raman spectroscopy. Moreover, several immune cells were studied under different activation/differentiation settings [10,11,12]. Recently, it was shown that the differentiation process of murine naïve T cells to effector T cells could be monitored [13].

This is, to the best of our knowledge, the first study to address how to distinguish CD3/CD28-stimulated living human T cell subsets (CD4/CD8) using Raman spectroscopy. Stimulating T cells serve to activate and promote their proliferation, closely mirroring the natural process that unfolds during an immune response within the body. The CD3/CD28 stimulation activates T cells by mimicking the co-stimulatory signals that occur during antigen presentation and is thought to be a more physiological approach than using phorbol myristate acetate (PMA) [14]. Furthermore, we analyzed the spectra of monocytes, another type of immune cell often associated with autoimmune diseases [15]. Knowing the unique Raman spectra for different immune cell conditions, such as stimulated T lymphocytes, is crucial for further studies aiming to identify the causes of autoimmune disease.

For this study, data were collected using a commercially available device known as CellTool BioRam. Raman spectroscopy was performed on optically trapped cells to enhance data acquisition, as freely moving cells can stray from the laser focus due to Brownian motion [16]. Notably, there is no need to chemically immobilize the cells, which could potentially alter their properties. Additionally, this technique reduces background fluorescence and reflections [17].

## 2. Results

**Figure 1:**
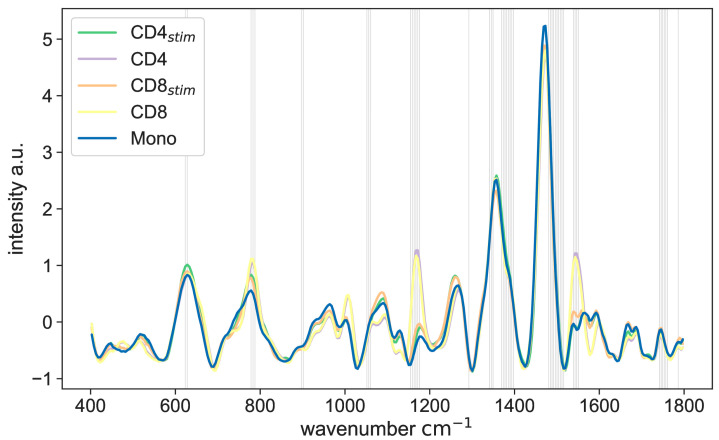
Raman spectra of CD4 cells, CD8 cells, and monocytes. Important differences between the spectra are marked with gray vertical lines. CD4: CD4+ T lymphocytes, CD8: CD8+ T lymphocytes, CD4_stim_/CD8_stim_: CD3/CD28-stimulated T lymphocytes, Mono: monocytes.

The results show that the Raman spectra of CD4 cells and CD8 cells are very similar but different from stimulated cells (Figure 1). In addition, the spectra of stimulated CD4 cells (CD4_stim_), stimulated CD8 cells (CD8_stim_), and monocytes show only minor differences.

It is interesting to note that these differences mainly occur for smaller and larger cells (Table A1). The identification of the discriminating bands will be further explained below by comparing two cell types.

**Figure 2:**
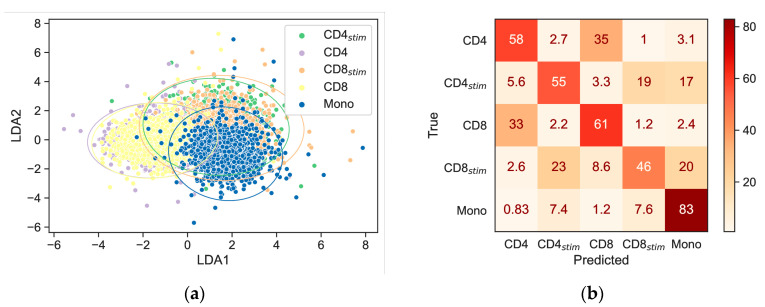
(**a**) Score plot for the linear discrimination analysis. (**b**) Aggregated confusion matrix from Leave-One-Out cross-validation normalized by true classes (row-wise, percentages). CD4: CD4+ T lymphocytes, CD8: CD8+ T lymphocytes, CD4_stim_/CD8_stim_: CD3/CD28-stimulated T lymphocytes, Mono: monocytes, LDA1/LDA2: 1st/2nd discriminant function.

For further analysis, as explained in the methods section, we carried out cross-validated Principal Component Analysis (PCA), followed by Linear Discriminant Analysis (LDA) (Figure 2a). The resulting aggregated confusion matrix is shown in Figure 2b. Monocytes can be distinguished best (83%). CD4 cells and CD8 cells are often confused with each other but are very unlikely to be recognized as a stimulated T cell or a monocyte. Similar applies to CD4_stim_ and CD8_stim_, which could only be distinguished as moderately reliable. Interestingly, they are quite frequently classified as monocytes.

**Figure A2:**
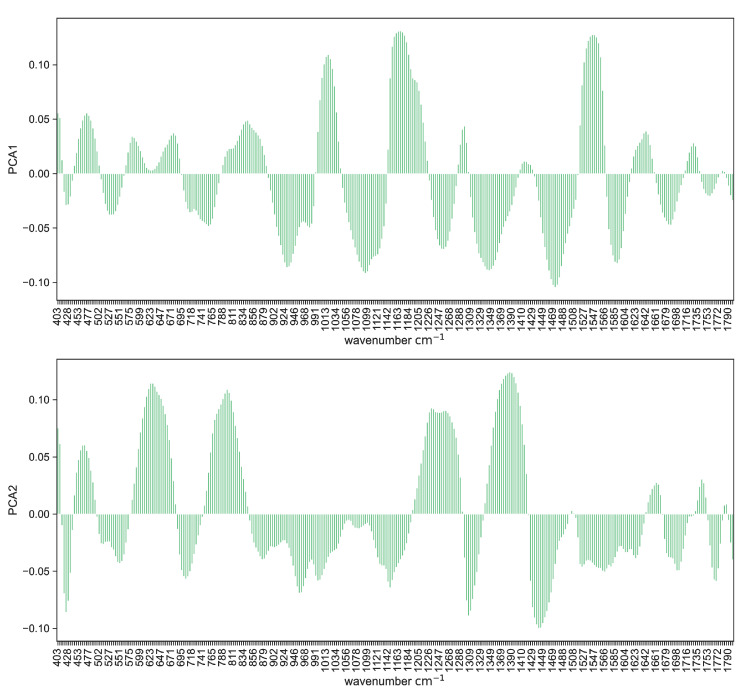
Exemplary representation of the loading vectors for the first two principal components. Every fifth wavenumber is shown.

In order to find specific discriminating immune cell characteristics, the analysis was carried out for two cell types each. We identified the most influential spectral features by analyzing the greatest loadings of the first principal components (Figure A2). Due to the complex molecular structure of cells, several molecules are involved in the discrimination of different cell types. The PCA/LDA analysis allows for the simplification of the complex Raman spectra with many overlapping peaks and subtle differences between cell types. Some of the most distinguishing features obtained from the loading vectors, as well as the discrimination accuracy, are listed in Table 1. The accuracy indicates the percentage of correctly classified cells calculated through Leave-One-Out cross-validation. Tentative molecular assignation to the discriminating bands is found in Table 2.

## 3. Discussion

In this report, we successfully acquired and analyzed the Raman spectra of more than 6500 immune cells in total from human donors. The recorded spectra were analyzed using PCA, followed by LDA. The results showed a striking difference between CD4 cells or CD8 cells and stimulated CD4 cells (CD4_stim_), stimulated CD8 cells (CD8_stim_), or monocytes (Table 2). This is in line with previous studies that revealed a clear difference between T cells and monocytes, or CD3/CD28-activated and inactivated T cells [7,11].

Our findings suggest that the difference results predominantly from two peaks (approximately 1160 cm^−1^ and 1550 cm^−1^). As optical trapping is known to mainly trap the cells by their nuclei, the differences should also mainly arise from the molecular composition of the nucleus [7,19]. However, we speculate that this might be due to the laser focus position inside the cell. It seems possible that larger cells (CD4_stim_, CD8_stim,_ and monocytes) are slightly displaced from the laser focus due to external forces (e.g., buoyancy and gravity). Consequently, the Raman spectrum of the cytoplasm or the nucleus may become more prominent, which are known to have fairly different Raman spectra [20,21].

CD4 and CD8, as well as CD4_stim_ and CD8_stim_, could be distinguished less precisely from each other. The accuracy was 63% and 70% for CD4/CD8 and CD4_stim_/CD8_stim_, respectively. This result is consistent with previous studies where CD4 and CD8 cells were discriminated with similar accuracy, likely due to the similarity of these cell types [8].

One limitation of this study is the absence of concrete evidence confirming the absence of phototoxic effects on the cells. Although we visually inspected the cells and did not find any signs of phototoxicity, the inability to definitively demonstrate the absence of such effects remains a limitation of our research.

We were able to show that Raman spectroscopy on stimulated immune cells could reliably detect subtle molecular differences. Simultaneously performing optical trapping helped keep non-adherent cells in focus and improved the optical measurement conditions by reducing reflections and fluorescence from the glass substrate. With a large number of measured cells, we believe we have provided a reliable database for further research. In particular, we demonstrated that T cell subsets (CD4, CD8) and monocytes could be well distinguished using Raman spectroscopy. Furthermore, we showed that stimulating the T cells with CD3/CD28 significantly alters the Raman spectrum, which then resembles the monocyte spectrum. However, there is uncertainty about the location of the laser focus within the cell and whether the differences arise solely from the nucleus. This is an issue that future research will need to explore. Nevertheless, our data suggest that Raman spectroscopy may have the potential for routine clinical diagnostics. Cellular biomarker monitoring is necessary to enable the profiling of individual disease characteristics, especially in autoimmune diseases. Raman spectroscopy has the potential to diagnose and monitor neuroimmunological and other inflammatory disorders. This could potentially lead to individualized treatments for specific patient groups identified by unique Raman signatures.

## 4. Materials and Methods

### 4.1. Cell Preparation

**Figure A3:**
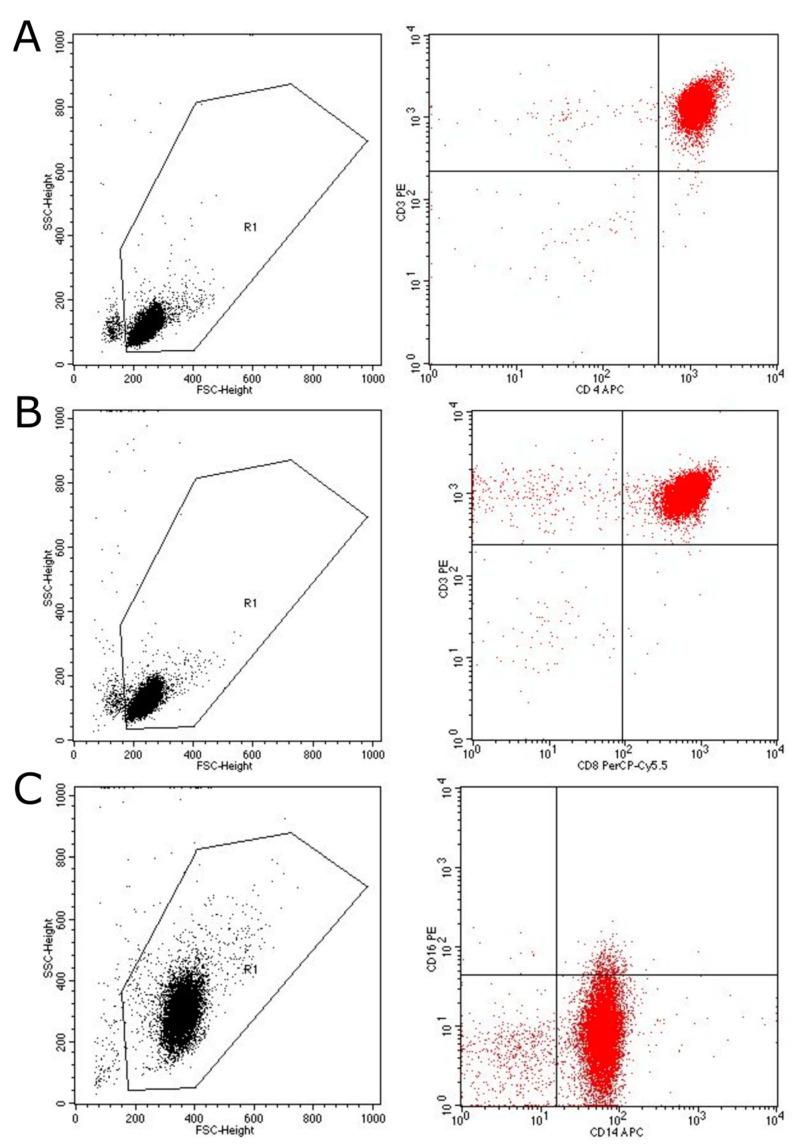
Representative flow cytometric dot plots depicting the purity of negatively selected cells. (**A**) Red dots in the upper right quadrant show the CD3 + CD4+ T cell population. They represent 96% of all cells. (**B**) Red dots in the upper right quadrant show the CD3 + CD8+ T cell population. They represent 94% of all cells. (**C**) Red dots in the lower right quadrant show the CD14 + CD16-monocyte population. They represent 90% of all cells.

Buffy coats from healthy donors were used to isolate cells (Table 3). The average age was 50.7 ± 12.1 years (mean ± SD), and 39.9% were female. First, peripheral blood mononuclear cells (PBMCs) were isolated by Pancoll human (PAN-Biotech, Aidenbach, Germany) density centrifugation. Following this step, the CD4 and CD8 cells, as well as the monocytes, were isolated using the appropriate Miltenyi isolation kit and an autoMACS^®^ Pro Separator (Miltenyi Biotec, Bergisch Gladbach, Germany) [22,23,24]. Cell isolation was carried out according to the manufacturer’s recommendations and constant laboratory conditions. Once the samples were isolated, cells were transferred to a PBS buffer for the final measurements. Flow cytometry analysis showed a high purity of >90% for CD4/CD8 cells and monocytes (Figure A3), respectively (anti-CD4 (Clone: RPA-T4, BioLegend), anti-CD8 (Clone: SK1, BioLegend, San Diego, CA, USA), and anti-CD14 (Clone: Clone M5E2, BD Biosciences).

**Figure 3:**
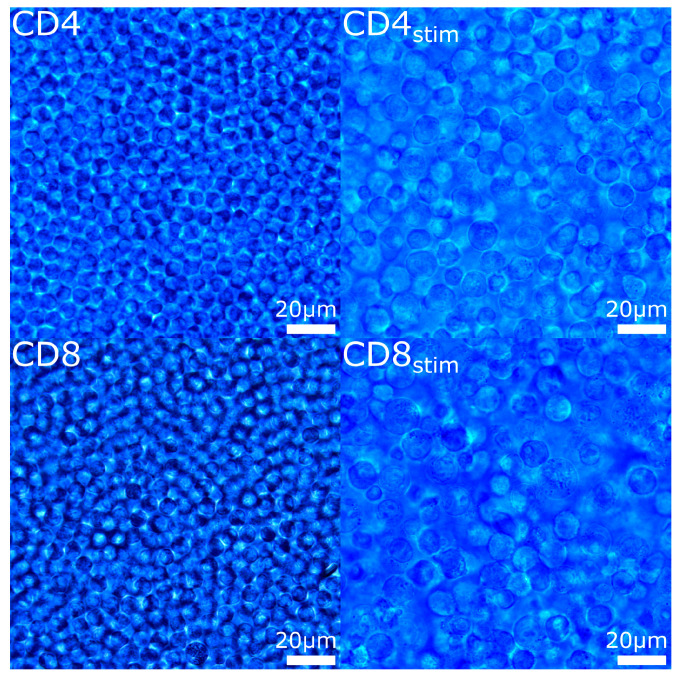
CD4 and CD8 cells before and after activation. CD4: CD4+ T lymphocytes, CD8: CD8+ T lymphocytes, CD4stim/CD8stim: CD3/CD28-stimulated T lymphocytes.

For the stimulation of CD4/CD8 cells, we utilized the ImmunoCult Human CD3/CD28 T Cell Activator (STEMCELL Technologies, Vancouver, BC, Canada). The kit contains antibodies that specifically target and activate the CD3 and CD28 co-stimulatory pathways on T cells. Cells were cultivated according to the manufacturer’s instructions for 72 h. Figure 3 depicts CD4 and CD8 cells both before and after activation. Afterward, the successful activation was verified via expression of the surface marker CD25 using flow cytometry analysis (anti-CD25 (Clone M-A251, BD Biosciences, Franklin Lakes, NJ, USA)). All methods were performed in accordance with the relevant guidelines and regulations.

### 4.2. Raman Spectroscopy

The principle of Raman spectroscopy is defined by the inelastic scattering of monochromatic light. When incident light interacts with a sample, a small fraction of photons undergoes a change in energy due to the interaction with molecular vibrations. These altered photons carry information about the vibrational modes of the molecules in the sample. The resulting Raman spectrum is a unique fingerprint that allows us to identify chemical compounds and investigate molecular changes [25].

**Figure 4:**
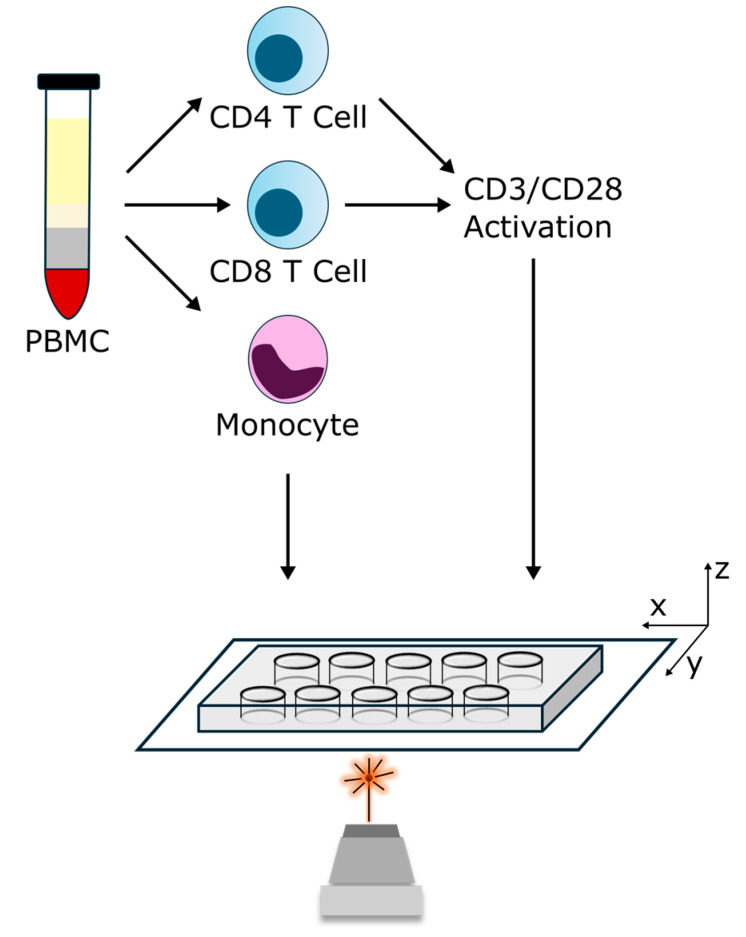
The schematic diagram shows the Raman spectroscopy setup, which includes an inverted microscope with a motorized sample platform. The Raman laser is directed onto the sample through the objective lens. Before analysis, CD4 cells, CD8 cells, and monocytes are isolated from peripheral blood mononuclear cells (PBMCs). Furthermore, CD4 and CD8 cells are examined in their activated state following CD3/CD28 stimulation.

Raman spectra were captured using a Raman spectroscopy device (CellTool BioRam, wavelength 785 nm, resolution approx. 4 cm^−1^). A 60× water immersion objective lens was used to focus the Raman laser onto the sample. Figure 4 provides a schematic representation of the experimental setup. Prior to data collection, cells were stored in a 10-well borosilicate glass bottom dish (Greiner bio-one, Kremsmünster, Austria). Approximately 2 × 10^6^ cells were required to create a monolayer at the glass bottom. Before the measurements started, the laser focus was manually aligned on the cell monolayer. To ensure that the signal originated from the cell itself, we performed reference measurements on the surrounding PBS. Subsequently, cells were automatically trapped at predefined randomly selected grid points (spacing 20 µm) of the monolayer at a height of about 20 µm above the glass bottom. The optical forces generated by the laser beam are used to trap and manipulate the cell. The balance between the scattering and gradient forces keeps the cell confined within the laser’s focal point (1–2 µm). Between measurements, the laser beam is briefly interrupted to trap the next cell. For each cell, 10 spectra were accumulated with a laser power of 80 mW and an exposure time of 3 s (Irradiance = 8 MW cm^−2^, Dose = 2.4 J). We required a total of about 35 s for the measurement of a single cell. After the measurement, visual inspection did not reveal any cell damage that might indicate potential phototoxic cellular impairment.

### 4.3. Data Analysis

The analysis was carried out with Python 3.9.8 and scikit-learn 1.0.1 [26]. Raman spectra were analyzed in their “fingerprint” region (400 to 1800 cm^−1^), which is considered to contain most of the biological information [27]. The averaged spectrum for a single cell was pre-processed using outlier removal, smoothing, baseline subtraction, and normalization. The outlier removal was performed by calculating the Z-scores for all data points within a spectrum. Spectra containing peaks with Z-scores greater than three (which corresponds to three standard deviations) were excluded from the analysis. For smoothing, a Savitzky–Golay filter with a window size of 19 and a polynomial degree of six was applied. The data were normalized by subtracting the mean and dividing by the standard deviation. Subsequently, for each cell type, a mean spectrum was calculated by the pre-processed data for all donors. Afterward, spectral data were analyzed using a Principal Component Analysis (PCA) followed by a Linear Discriminant Analysis (LDA). The number of principal components was selected to achieve an explained variance greater than 90%. For the LDA comparing two cell types, we performed dimensionality reduction to one component using the eigenvalue solver and applied shrinkage based on the Ledoit-Wolf lemma. We used the Leave-One-Out method for cross-validation. An aggregated confusion matrix was calculated by combining the individual confusion matrices from each iteration of Leave-One-Out cross-validation and summing them. Row-wise normalization was then applied by dividing each row by the sum of its values, highlighting the proportion of correctly and incorrectly classified instances for each class. The average cell diameter was estimated from 15 cells using Fiji 2.3.0 (Table A1).

## Figures and Tables

**Table 1:** Discrimination accuracy of different immune cells by their Raman signals.

Compared Cell Types	Accuracy (Mean ± SD)	Approx. Discriminating Bands ^1^ (cm^−1^)
CD4:CD8	0.63 ± 0.48	1167, 1546, 1445
CD4:CD4_stim_	0.92 ± 0.28	1163, 1546, 1389
CD8:CD8_stim_	0.91 ± 0.29	1163, 1546, 628
CD4_stim_:CD8_stim_	0.7 ± 0.46	1385, 623, 1457
CD4:Monocytes	0.96 ± 0.19	1167, 1546, 1385
CD8:Monocytes	0.96 ± 0.19	1167, 1546, 1389
CD4_stim_:Monocytes	0.83 ± 0.37	1385, 1464, 623
CD8_stim_:Monocytes	0.82 ± 0.38	1385, 623, 1154

^1^ Estimated by the highest loadings of the first principal components.

**Table 2:** Tentative molecule assignation.

Approx. Discriminating Bands (cm^−1^)	Tentative Assignation (Examples) [18]
1167	C=N stretching in a quinoid ring, C-H in the plane, Tyrosine, Lipids
1546	NADH, C6-H deformation mode, Tryptophan, Amide II
1445	δ (CH2), δ (CH3), phospholipids
1163	Tyrosine, quinoid ring
1389	CH rocking, C-N stretching
628	Glycerol, Methionine
1385	CH3 band, CH rocking
623	Proteins
1457	Deoxyribose, Nucleic acids, proteins

δ—bending mode.

**Table 3:** Sample characteristics.

Cell Type	Number of Donors	Number of Measured Cells
CD4	16	1637
CD8	15	1687
CD4_stim_	9	961
CD8_stim_	8	856
Monocytes	14	1453

**Figure A1:**
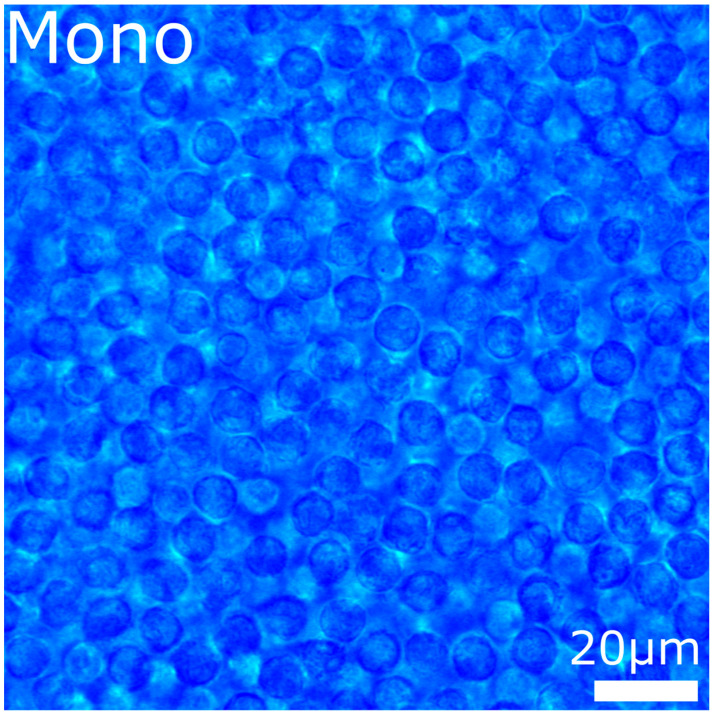
Monocytes.

## Data Availability

The data presented in this study are available on request from the corresponding author due to ethics approval.

## Appendix A

**Table A1:** Average cell diameter estimated from 15 cells for each cell type.

Cell Type	Diameter (Mean ± SD, µm)
CD4	6.6 ± 0.5
CD4_stim_	10.9 ± 1.4
CD8	6.8 ± 0.6
CD8_stim_	11.3 ± 2.2
Mono	8.5 ± 0.4
